# Supplementary material for: Genetic variation and RNA structure regulate microRNA biogenesis
Source: Nat Commun. 2017 May 3;8:15114. doi: 10.1038/ncomms15114 (PMC5418625; doi:10.1038/ncomms15114)
Supplement: Supplementary Information — Supplementary Figures and Supplementary Table [file ncomms15114-s1.pdf]

Supplementary Material

Supplementary Figures

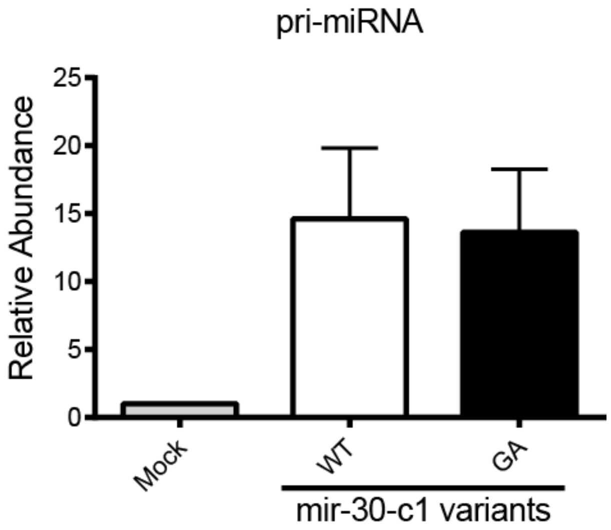

Supplementary Figure 1 | Relative expression levels of primary miRNAs, pri-miR-30c-1 WT and the GA variant in MCF7 cells. Error bars indicate SEM (n=5)

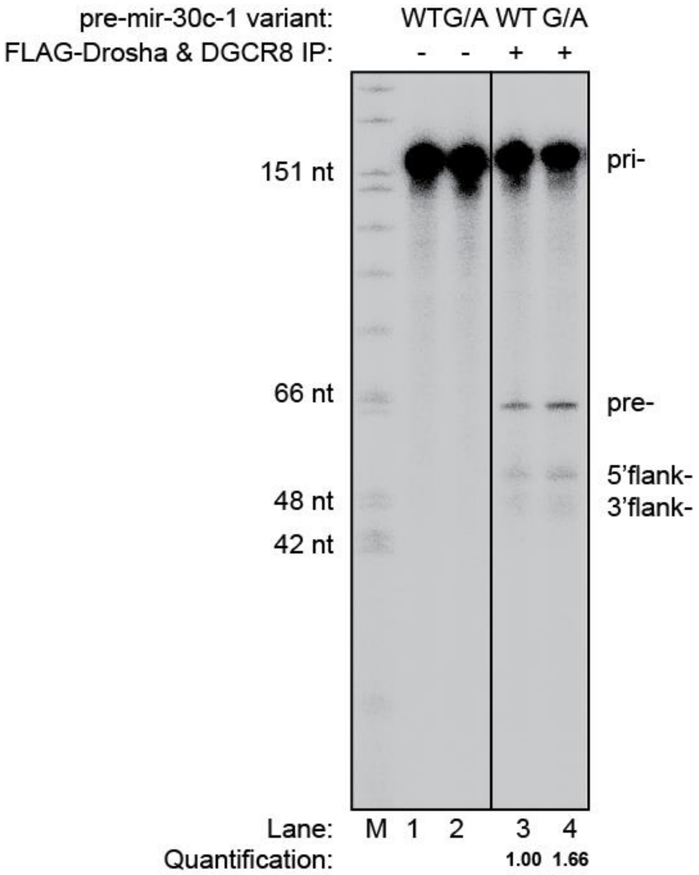

Supplementary Figure 2 | Representative *in vitro* processing of shorter pri-mir-30c-1 substrates (153nt) comprising either a wild-type sequence (WT) or bearing the G/A mutation in the presence of immunopurified FLAG-Drosha/FLAG-DGCR8 complexes (+) or FLAG imunoprecipitates (-) from HEK293T cells. Quantification of pre-miRNA band intensities are shown below and expressed as the relative intensity normalized to pre-mir-30c-1 WT variant (lane 3). Intervening lanes were removed from the gel image, which is indicated by the black vertical line.

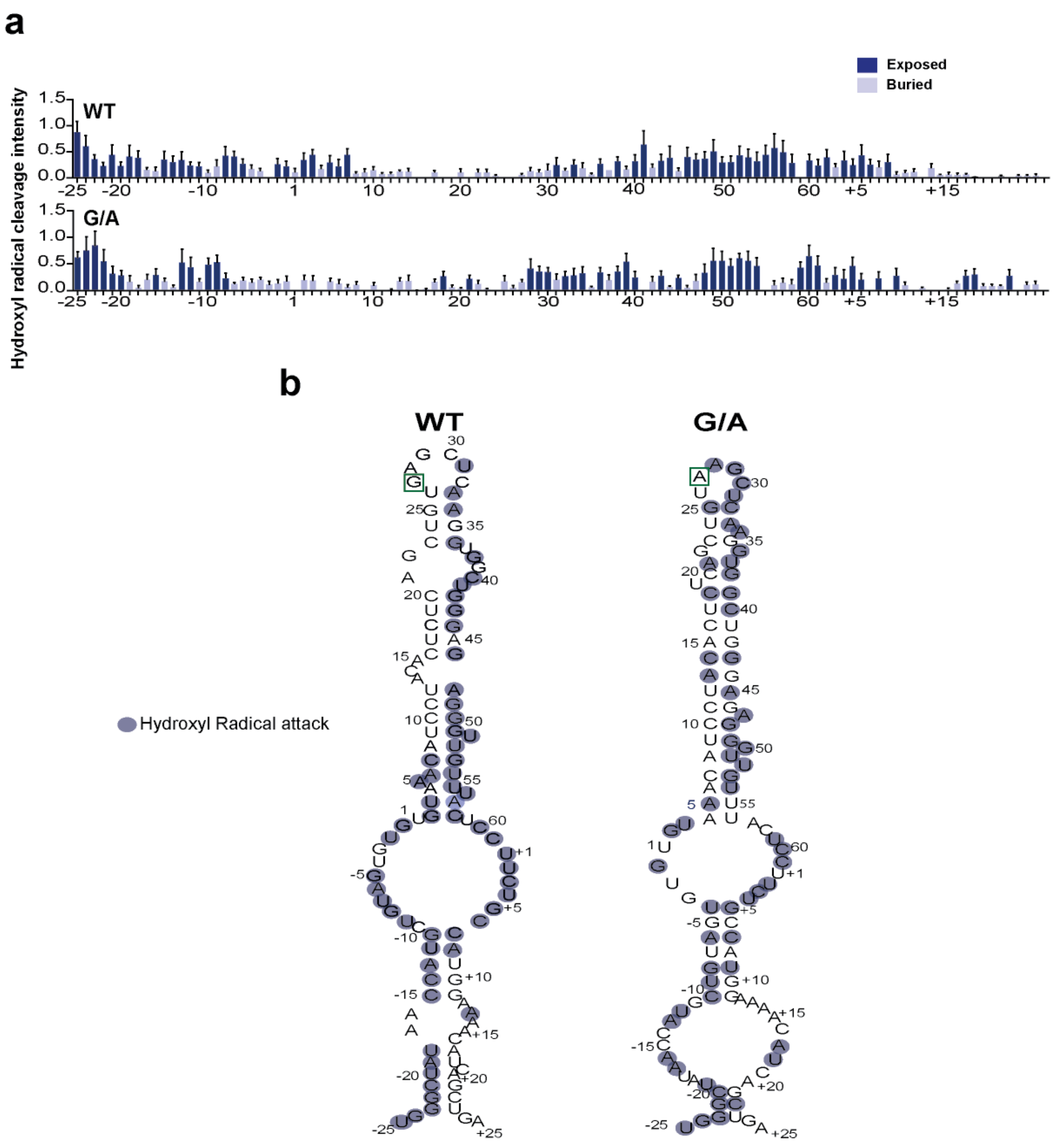

Supplementary Figure 3| (**a**) Histograms of cleavage intensity versus nucleotide position determined using *in situ*-generated hydroxyl radicals values corresponding to the mean cleavage (+/- SEM) (n=6). (**b**) Predicted secondary structures of WT and G/A variant sequences, as determined by hydroxyl radical reactivity values.

a

	Accession number	Kda	Identified protein	Number peptides (WT/GA)
COMMON	P11142	71	Heat shock cognate 71 Kda protein	10/8
	P09651	39	Heterogeneous nuclear ribonucleoprotein A1	5/8
	P09874	113	Poly [ADP-ribose] polymerase 1	5/3
	Q00839	89	Heterogeneous nuclear ribonucleoprotein U	5/4
	Q92841	80	ATP-dependent RNA helicase DDX17	5/4
	P52597	46	Heterogeneous nuclear ribonucleoprotein F	3/4
	P22626	37	Heterogeneous nuclear ribonucleoproteins A2/B1	3/6
WT	P35637	53	RNA binding protein FUS	3
	P38646	70	stress 70 protein mitochondrial	3
	P50991	57	T complex protein 1	3
	Q16778	14	Histone H2B type 2E	3
	Q96RS2	33	40S ribosomal protein	3
G/A	P84103	20	Serine/arginine-rich splicing factor 3	3

b

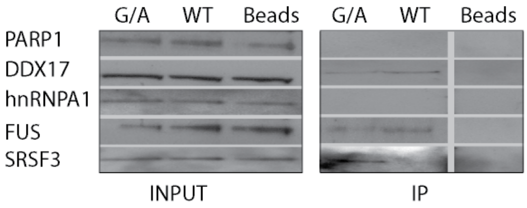

Supplementary Figure 4 | Identification of proteins interacting with WT and G/A pri-miR30c-1 sequences. (**a**) Table shows proteins identified in two independent experiments. (**b**) Validation of the interaction of SRSF3 with the G/A variant sequence by RNA immunoprecipitation followed by Western Blot analysis with specific antibodies.

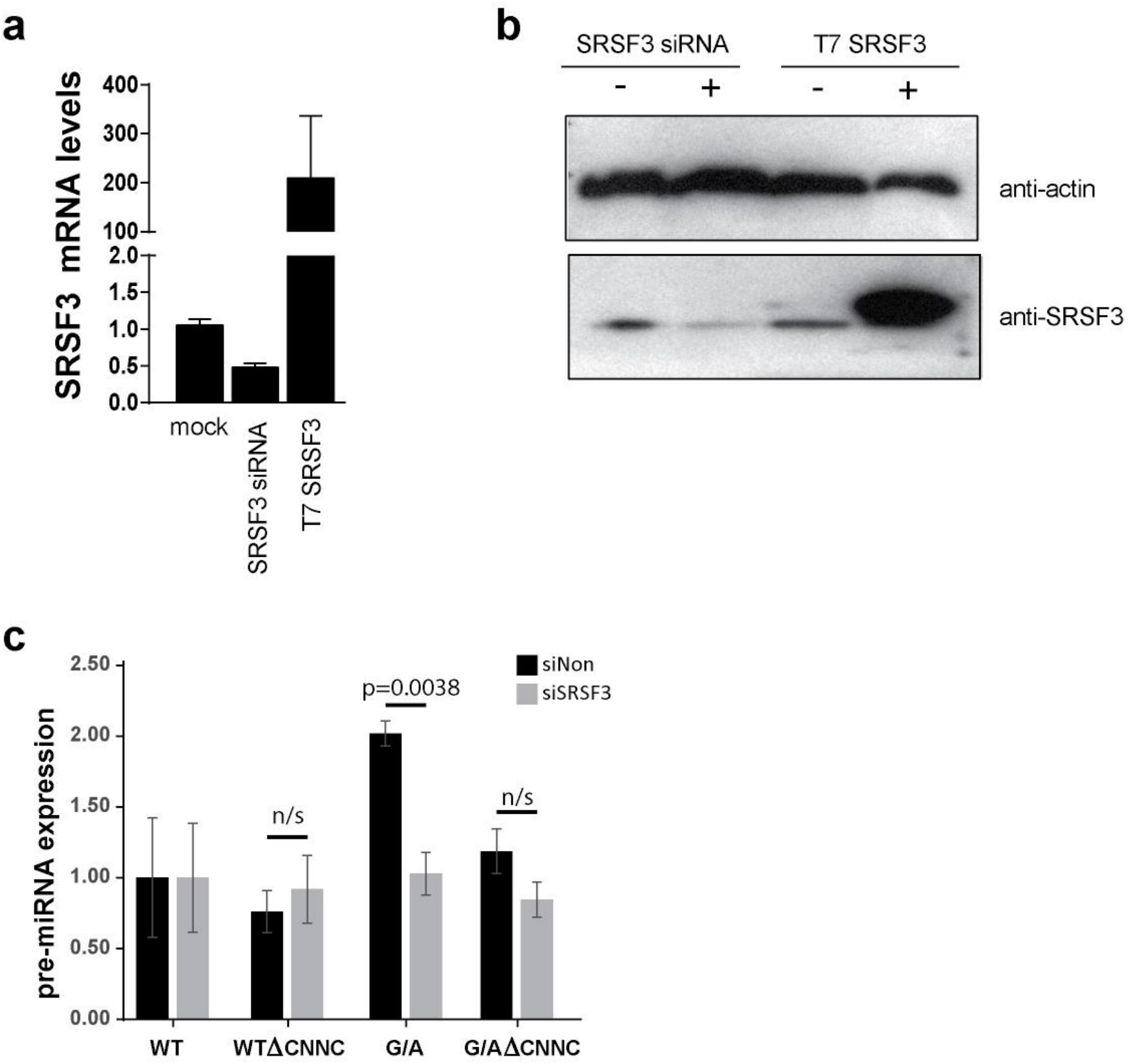

Supplementary Figure 5 | SRSF3 levels following knock-down or overexpression in MCF7 cells. (**a**) Analysis of SRSF3 mRNA levels. Error bars indicate SEM (n=5). (**b**) Representative Western blot of SRSF3 protein levels. (**c**) Relative expression levels of precursors, pre-miR-30c WT, WT ΔCNNC, G/A and G/A ΔCNNC in HeLa cells upon knock-down of SRSF3 (siSRSF3), as compared to a control siRNA (siNon). Pri- and Pre- levels were calculated using primers housed within the primary and precursor sequence of miR30c. The graph shows the relative levels of pre-miR30c normalized to either the siNon or siSRSF3 miR30c-1 WT. Significance between siNon/siSRSF3 miR30c levels was calculated using a t-test. Error bars indicate SEM (n=3)

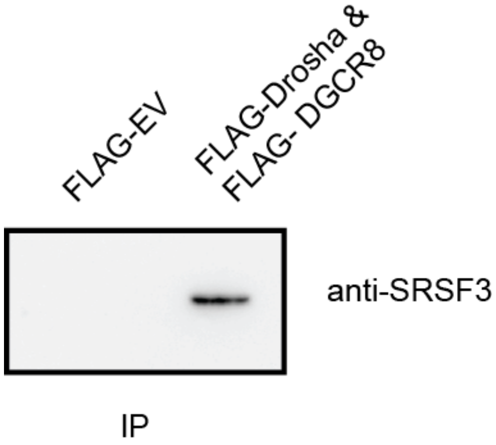

Supplementary Figure 6 | Detection of SRSF3 protein in FLAG-Drosha/FLAG-DGCR8 complexes used in the *in vitro* processing assays shown on Fig 6 and Supplementary Fig. S2.

a

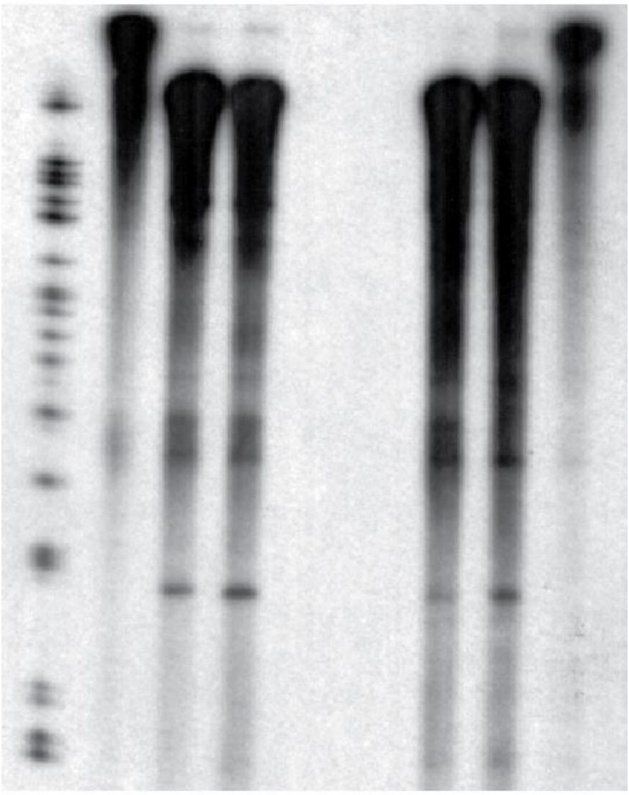

b

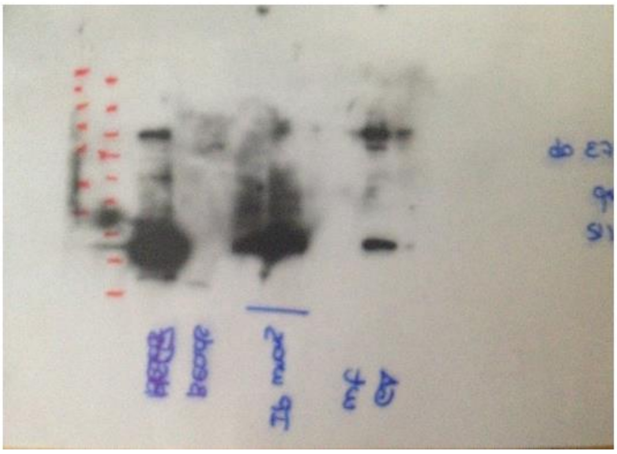

Supplementary Figure 7 | Uncropped scan Figures (**a**) Uncropped Exposure for Figure 1 d (**b**) Uncropped Western Blot for Figure 4 b.

Supplementary table 1 | Oligonucleotide sequences

Name	Sequence 5´-3´	Orientation	modification
**External**			
30c-1s	CAAGTGGTTCTGTGTTTTTATTG	Forward	
30c-1a	GTACTTAGCCACAGAAGCGCA	Reverse	
CNNCs	CTGCTTACTGGCTTATCG	Forward	
CNNCa	GATATCTGCAGAATTCACTAG	Reverse	
**mutagenics**			
30Cmut1	CCTTGAGCTTACAGCTGAGAG	Reverse	
30Cmut2	CTCTCAGCTGTAAGCTCAAGG	Forward	
CNNCmut1	CTTCATTTGATGTTTTCCATGGC	Reverse	
CNNCmut2	CTTCTTTTTTTTTTTCCATGGC	Reverse	
DCNNC	CTTCAGATGTTTTCCATGGC	Reverse	
**RT-qPCR**			
TL	CTGCTTACTGGCTTATCG	Forward	
TL	CCACTTGAATCGAATTCCAG	Reverse	
b-actin	GGGTCAGAAGGATTCCTATG	Forward	
b-actin	GGTCTCAAACATGATCTGGG	Reverse	
**Primer extension**			
premir30c1 RT	CTAGATGCATGCTCGAGCG	Reverse	NED
premir30c1 RT	CTAGATGCATGCTCGAGCG	Reverse	VIC
**RNA oligos**			
Pre-miR-30c wt	UGUAAACAUCCUACACUCUCAGCUGUGAGCUCAAGGUGGCUGGGAGAGGGUUGUUUACUCC		
Pre-miR-30c G/A	UGUAAACAUCCUACACUCUCAGCUGUAAGCUCAAGGUGGCUGGGAGAGGGUUGUUUACUCC		
Pre-miR-30a	UGUAAACAUCCUCGACUGGAAGCUGUGAAGCCACAAAUGGGCUUUCAGUCGGAUGUUUGCAGC		
